# A Large-Scale Multi-Hop Localization Algorithm Based on Regularized Extreme Learning for Wireless Networks

**DOI:** 10.3390/s17122959

**Published:** 2017-12-20

**Authors:** Wei Zheng, Xiaoyong Yan, Wei Zhao, Chengshan Qian

**Affiliations:** 1 School of Computer Engineering, Jinling Institute of Technology, Nanjing, 211169, China; zhengwei@jit.edu.cn (W.Z.); zhaowei@jit.edu.cn (W.Z.); 2 School of Modern Post & Institute of Modern Posts, Nanjing University of Posts and Telecommunications, Nanjing 210003, China; 3 School of Computer & Software, Nanjing University of Information Science and Technology, Nanjing 210044, China; qianchengshan@nuist.edu.cn

**Keywords:** larger-scale wireless multi-hop localization, regularized extreme learning, machine learning

## Abstract

A novel large-scale multi-hop localization algorithm based on regularized extreme learning is proposed in this paper. The large-scale multi-hop localization problem is formulated as a learning problem. Unlike other similar localization algorithms, the proposed algorithm overcomes the shortcoming of the traditional algorithms which are only applicable to an isotropic network, therefore has a strong adaptability to the complex deployment environment. The proposed algorithm is composed of three stages: data acquisition, modeling and location estimation. In data acquisition stage, the training information between nodes of the given network is collected. In modeling stage, the model among the hop-counts and the physical distances between nodes is constructed using regularized extreme learning. In location estimation stage, each node finds its specific location in a distributed manner. Theoretical analysis and several experiments show that the proposed algorithm can adapt to the different topological environments with low computational cost. Furthermore, high accuracy can be achieved by this method without setting complex parameters.

## 1. Introduction

An important issue of concern after monitoring an event or information is the location of the event or the collection of information in a variety of wireless network applications [1,2,3], such as the area of the enemy’s movement on the battlefield, the location of forest fires, etc., which is the foundation of further measures and decision-making. Due to cost, lack of indoor signals and other reasons, it is difficult to lay GPS localization modules on wireless nodes. Therefore, the wireless network needs a localization algorithm which conforms to its own characteristics, and obtains the exact location of the unknown node through communication between the nodes.

Depending on the different focus, localization algorithms can be divided into various types [4,5], such as, on the basis of whether it is necessary to measure the distance information between nodes, localization algorithms can be divided into range-based and range-free; or according to the localization process, localization algorithms can be divided into centralized computing and distributed computing. In the practical application, various demands invoke different algorithms, in the light of their quality, stability, and efficiency. The range-free algorithm is mainly based on the connectivity of the network and the information fed back by the neighboring nodes. The unknown node calculates its own estimated position. The range-free algorithm has advantages of low network construction cost and small communication cost, and distributed computing has advantages of small computational burden and insensitivity to the network scale. If the complex environment and the node size of the monitoring area are taken into account, distributed computing can better adapt to the real needs. Therefore, the range-free algorithm is highly suitable for large-scale wireless network localization [6]. However, as with many key technologies, the range-free multi-hop localization algorithm is still plagued by technical problems in practical application. The most problematic is that multi-hop localization can only be achieved in an isotropic network with high node density and uniform distribution, while in the anisotropy network such as nonuniform distribution of nodes or sparse, the localization effect is very poor [4,7,8,9].

**Figure 1:**
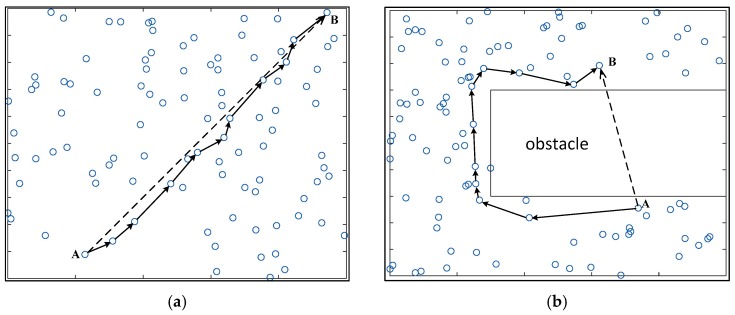
Different distribution of nodes (**a**) Isotropic network; (**b**) Anisotropic network.

Anisotropy indicates that the quality of physical properties has different values when measured along axes in different directions. Anisotropy is most easily observed in the wireless network. As shown in Figure 1, we use $d_{AB}$ (the dashed line) to represent the physical distance between nodes A and B in the network, and use $h_{AB}$ (the straight line with arrows) to indicate the approximate length of the shortest hop-counts between nodes A and B. When the nodes are isotropic and uniformly distributed in the network, we can see that $d_{AB}\propto h_{AB}$; however, when there is an “obstacle” that affects the signal transmission in the network, $d_{AB}<<h_{AB}$. Obviously, if no “obstacle” is present in the network, the physical distance between the nodes is proportional to the hop-count between them, that is $d_{AB}\propto h_{AB}$. When there is an obstacle in the network, this proportional relationship is no longer true. This paper focuses on handling anisotropic network problems in large scale multi-hop localization.

For the problems existing in the multi-hop localization, this paper presents a novel multi-hop localization method, called Multi-hop Localization though Regularized Extreme Learning Machine (ML-RELM, which is more suitable for practical applications because of its relatively lower computational complexity, higher localization accuracy, and stronger adaptability. The ML-RELM method aims to improve the performance of the localization algorithm and to adapt it to different environments by using the topology of the wireless network and its own characteristics.

## 2. Related Work

The most well-known multi-hop distributed localization algorithm is the DV-hop proposed by Niculescu and Nath [10]. In the DV-hop method, the distances between the locating nodes to the anchor nodes can be represented by the product of network average hop distance and the hop-counts between them, then to obtain the location information via trilateration. For example, the average hop distance of anchor i can be calculated by Equation (1):

(1) $$c_{i}=\frac{\sum_{i\neq j}d_{ij}}{\sum_{i\neq j}h_{ij}}$$

where $d_{ij}$ is the physical distance between anchor node i and anchor node j, and $h_{ij}$ is the corresponding shortest hop-count. The DV-Hop algorithm is simple and easy to implement. The whole localization process does not rely on additional hardware devices. However, its adaptability to the environment is poor, therefore the applications are limited.

By virtue of machine learning methods, modeling and designing the localization mechanism has become a hot topic in recent years [11,12]. Based on the correspondence between the distributed characteristics and the measured information of the known nodes, a mapping model can be learned. Then, this model is used to estimate the position of the unknown nodes. Compared with the previous work, such model can effectively find out the intrinsic network topology, the relevance of observations and other information hidden in large-scale data, which then effectively improve the localization performance. Lim et al. [9] proposed a Proximity Distance Map (PDM) localization algorithm using Truncated Singular Value Decomposition (TSVD) technology to model hop-counts and physical distance [13]. The PDM method first records the physical distance and the measured distance between the known nodes with the matrix respectively. Secondly, it transforms the two matrices linearly through the TSVD method to obtain an optimal linear transformation model. Thirdly, it takes the shortest hop-count from unknown nodes to anchor nodes into the model. Finally, the physical distance of unknown nodes to anchor nodes is estimated. The TSVD is essentially a linear multivariate rule by which the estimated distance obtained is the weighted sum of the estimated values of the known nodes associated with the unknown nodes, and thus the estimated values are closer to the true values. In addition, TSVD drops the smaller singular values. To a certain extent, this operation reduces the impact of noise in the conversion process, so as to enhance the stability of the algorithm. However, the PDM method ignores the problem of scale conversion between the hop-counts matrix and the physical distances matrix, which can cause data loss. In addition, it does not offer a choice for TSVD clip coefficients. Accordingly, an excessive coefficient may easily cause useful information lost, while a too small clip coefficient would retain too much high-frequency noise. 

Recently, inspired by the PDM method, Lee and others proposed multi-hop range-free localization methods based on SVM [14,15], namely, Localization through Support Vector Regression (LSVR) and Localization through Multi-dimensional Support Vector Regression (LMSVR) [7,8]. These two methods suggest that there is a nonlinear relationship between the hop-counts and the physical distances. They use a regression based on the kernel technology SVM to predict the distance between nodes. However, according to Occam’s razor principle, the more complex the model, the higher the probability of over-fitting [16]. Therefore, the localization method based on SVM algorithm has the over-fitting phenomenon with the increase of the number of anchor nodes, which resulted in the reduction of the localization accuracy. In addition, SVM-based methods are prone to excessive computational complexity.

**Figure 2:**
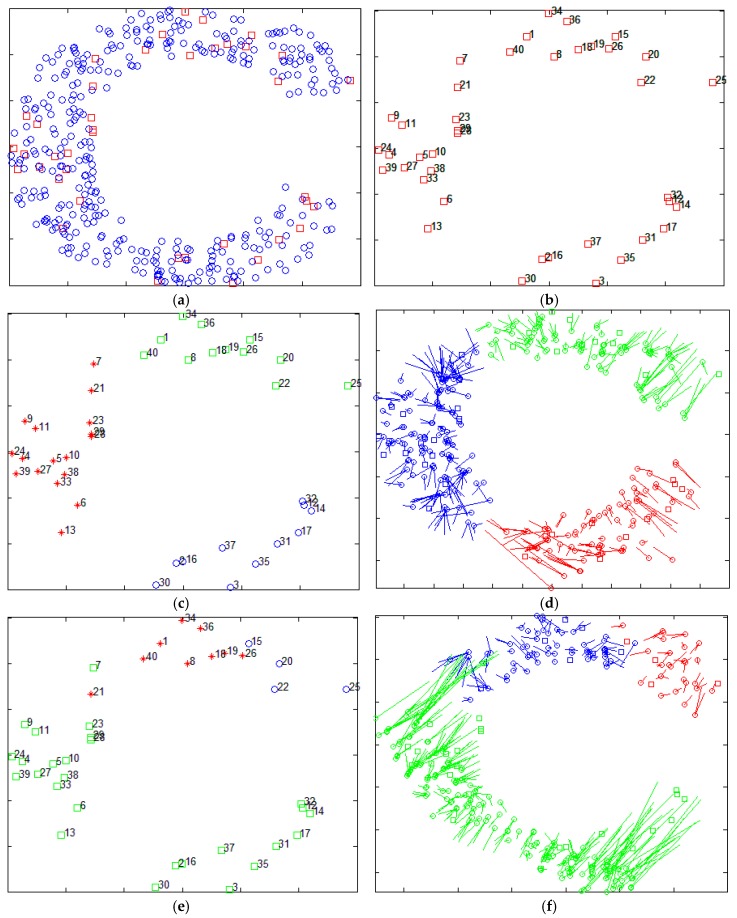

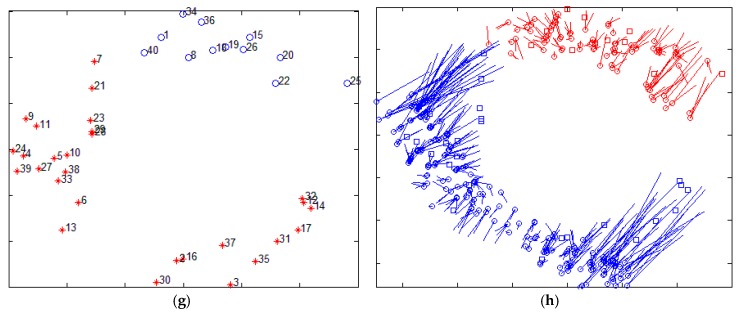
The localization results of SM for different regional center nodes and for different number of regions (**a**) The distribution of nodes; (**b**) The distribution of anchor nodes; (**c**) The center node is #12, area is divided into three parts; (**d**) The localization result of SM corresponds to the Figure 2c; (**e**) The center node is #17, area is divided into three parts; (**f**) The localization result of SM corresponds to the Figure 2e; (**g**) The center node is #12, area is divided into two parts; (**h**) The localization result of SM corresponds to the Figure 2g.

The Xiao team from Hong Kong Polytechnic University put forward another strategy. They proposed a series of distributed multi-hop localization algorithm, such as Pattern-Driven localization Scheme (PDS) [17], Reliable Anchor-based Localization (RAL) [18], Anchor Supervised (AnSuper) [19], and Selective Multilateration (SM) [20]. Based on the anchor node information, the family of algorithms divides the deployment area into a number of uniformly distributed set of nodes. Taking the SM algorithm as an example, it uses the angle between the anchor nodes to measure the distance between the anchor nodes and divide the area. However, the SM method also requires human intervention in the selection of the number of divided regions and the regional center nodes. Figure 2 shows the localization results of the SM algorithm, which selects different regional centers and the number of regions. The figure randomly distributes 400 nodes (as shown in Figure 2a), of which 40 are anchor nodes (shown in Figure 2b). Figure 2c,e,g are the anchor nodes partition map, and Figure 2d,f,h are the corresponding localization results. Figure 2d,h are centered on node 12 and the nodes are divided into three and two areas, respectively. Figure 2f is centered on node 17 and the area is divided into three regions. It is easy to find, with different nodes as the center and divided into different numbers of regions obtained by localization, that results are not the same, which confirms the instability of the algorithm.

The latest study based on the number of packets between nodes and estimated distance residuals, uses Multi-objective Optimization to carry out the node position estimation [21,22]. But the bionic methods employed in the localization algorithm use pseudo-random number, so the results are not same each time. In addition, the optimal solution requires multiple iterations, which affects the efficiency of the algorithm.

The Extreme Learning Machine (ELM) is a new learning method of single-hidden-layer feedforward neural networks (SLFNs) proposed by Huang [23]. In the ELM, the input weights (connecting the input nodes to the hidden nodes) and the hidden layer nodes are randomly selected, and the output weights (connecting to the output node of hidden layer node) are determined by calculating the Moore-Penrose (MP) generalized inverse of the hidden layer output matrix. This learning algorithm ensures the generalization performance of the network, and significantly enhances the convergence as well. Moreover, the additional consumption on the tunes for learning rates, and the curses of local minimizers can be avoided [24]. Because of satisfactory precision and high efficiency, ELM has a very wide application. In this paper, a new distributed multi-hop method, ML-RELM, is proposed by combining ELM and distributed multi-hop localization algorithm. This method constructs the optimal relation (model) by the information of the actual distance and the number of inter-node hops, and then uses this model to predict the distance between the unknown node and the known node.

## 3. Regularized Extreme Learning 

**Figure 3:**
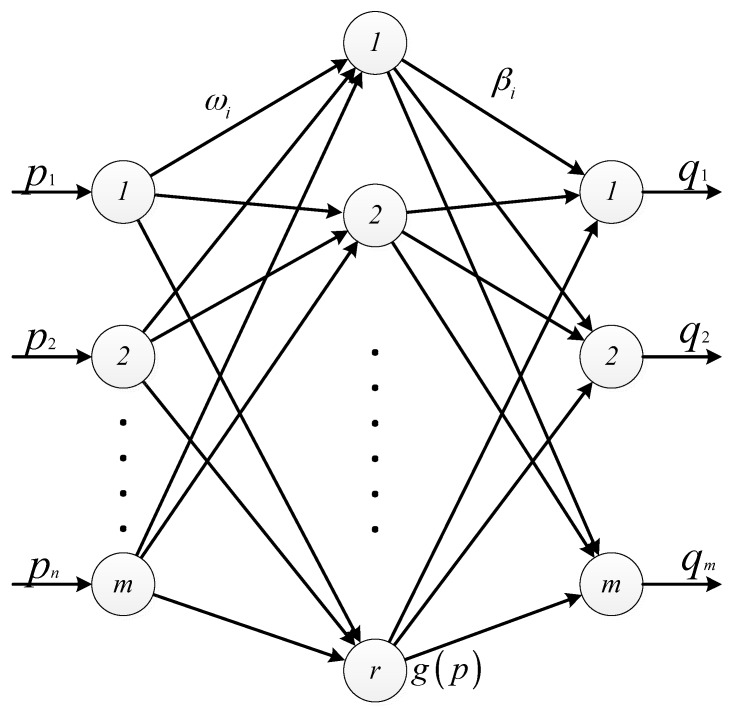
The schematic diagram of ELM.

Huang et al. [23] proposed in 2006 a learning method based on Single Hidden Layer Feedforward Neural Networks (SLFNs) that is an Extreme Learning Machine. The main idea is to randomly assign the input weight and hidden layer deviation, to set the hidden layer of the number of nodes, and then through the least squares method to determine the value of the output weight. ELM algorithm overcomes the shortcoming of local minimization and over-fitting in traditional gradient-based neural network algorithms (such as BP algorithm). Thus, the ELM is a quick speed and less intervention single hidden layer feedforward neural network. It has excellent ability of regression. In addition, ELM does not need to manually set and adjust the parameters of the classifier, the completion of the entire process without iteration. Comparing to traditional learning methods, it is easier to deploy and implement. The basic principle of the ELM is shown in Figure 3.

Figure 3 shows a single hidden layer feedforward neural network. Among the network there are *m* input nodes, *k* hidden layer nodes, *m* output nodes; ***ω****_i_* is the input weight vector of the *i* hidden layer nodes, which represents the weight input of the *j* input node to the *i* hidden node; *b_i_* is the deviation of the *i* hidden layer node (or the influence factor); ***β****i* is the weight value of the *i* hidden node output to the *j* output node; *g*(*p*) is the activation function. For the activation function, Huang et al. have proved that it is an infinitely differentiable non-constant function. ELM has the characteristic of the full rank, thus it guarantees the accuracy of ***ω*** and ***b*** randomly assigned. 

For any given input data set (***U,V***) = {(***u***_1_,***v***_1_),(***u***_2_,***v***_2_)…(***u****_n_*,***v****_n_*)}, the input of the *i* sample is ***u****_i_* = [*u_i_*_1_,*u_i_*_2_,…,*u_im_*]^T^ ∈ *R^m^*, and the output is ***v****_i_* = [*v_i_*_1_,*v_i_*_2_,…,*v_im_*]^T^ ∈ *R^m^*. When the *i* sample is input to the ELM, the input of the *j* hidden layer node is shown as Equation (2):

(2) $$\begin{array}{l} \omega_{j}u_{i}=\left(\omega_{j1},\omega_{j2},\cdots ,\omega_{jn}\right)\left(\begin{array}{c} u_{i1}\\ u_{i2}\\ \vdots \\ u_{im} \end{array}\right)\\ \;\;\;=\omega_{j1}u_{i1}+\omega_{j2}u_{i2}+\cdots +\omega_{jm}u_{im}. \end{array}$$

After the processing of the *j* hidden layer node, the output is $g\left(\omega_{j}u_{i}+b_{j}\right)$. At this time, the output matrix with *r* hidden nodes is:

(3) $$G\left(u_{i}\right)={\left(g\left(\omega_{1}u_{i}+b_{1}\right),g\left(\omega_{2}u_{i}+b_{2}\right),\cdots ,g\left(\omega_{r}u_{i}+b_{r}\right)\right)}^{T}$$

According to the hidden layer output matrix $G\left(X\right)=\left(G\left(u_{1}\right),\ldots ,G\left(u_{r}\right)\right)$ and the output weight matrix ***β***, the output matrix ***V*** can be approximated as:

(4) $$\begin{array}{l} G{\left(u_{i}\right)}^{T}\beta =G{\left(u_{i}\right)}^{T}\left(\beta_{1},\ldots ,\beta_{m}\right)\\ =\left(g\left(\omega_{1}u_{i}+b_{1}\right),g\left(\omega_{2}u_{i}+b_{2}\right),\cdots ,g\left(\omega_{r}u_{i}+b_{r}\right)\right)\left(\begin{array}{ccc} \beta_{11} & \cdots & \beta_{m1}\\ \vdots & \ddots & \vdots \\ \beta_{1r} & \cdots & \beta_{mr} \end{array}\right)\\ =\left(o_{i1},\cdots ,o_{im}\right), \end{array}$$

where element *β_ij_* of the output matrix ***β*** represents the impact of the *j*-th node on the physical distance of the *i*-th node. The diagonal element *β_ii_* of the output matrix ***β*** can be regarded as the scaling factor when the hop-count is converted to distance. Therefore, the distance from the unknown node to the anchor node obtained by the extreme learning method can be seen as the weighted sum of the hop-counts with all anchor nodes.

The activation function of the ELM is infinitely differentiable and can infinitely approximate to the output value, which means the estimated output value with the zero error. Then there are suitable, that make $\overset{m}{\underset{j=1}{\sum }}\left|o_{i}-t_{ij}\right|=0$. So the least squares method can be used to obtain the output matrix ***β***:

(5) $$\beta =G^{†}V,$$

where $G^{†}$ is the Moore-Penrose (MP) generalized inverse matrix of the hidden weight output matrix ***G***, and ***V*** is the output matrix.

Furthermore, for multi-hop localization, the element of the hop-count matrix is mostly integer, which can cause the linear correlation between the vectors of the hop-count matrix, and the ill-conditioned problem may occur when solving the generalized inverse matrix ***G***^†^ (i.e., ***G***^†^ = (***G****^T^**G***)^−1^ ***G****^T^*). Therefore, we add a positive matrix *k**I*** (*k* > 0) as the regular term to the matrix (***G****^T^* ***G***)^-1^ , so that the result (***G****^T^**G*** + *k**I***)^−1^ will be not singular. It effectively improves ELM’s ability to deal with regression problems of linearly related variables, where *k* is a regular parameter, and ***I*** is the unit matrix. 

## 4. Distributed Multi-Hop Localization Based on Regularized Extreme Learning

The classical large-scale multi-hop localization methods assume that the network is isotropic and evenly distributed, so the hop-counts can correspond to the physical distance. Unfortunately, in practice, networks often present anisotropy. If a network is anisotropic, the hop-counts between nodes may not match the physical distances well. Using fixed coefficients (such as the DV-hop algorithm) to match the hop-counts and the physical distances will produce a greater error. Therefore, this match should be related to the direction and position. In order to solve this problem, we use regularized extreme learning to construct the model between the hop-counts and the physical distances, representing the anisotropy of the network. With the help of regularized extreme learning, the model describes the optimal linear transformation between hops and physical distances. With the help of the proposed algorithm, an unknown node can get a more accurate distance conversion, resulting in better position estimation.

**Figure 4:**
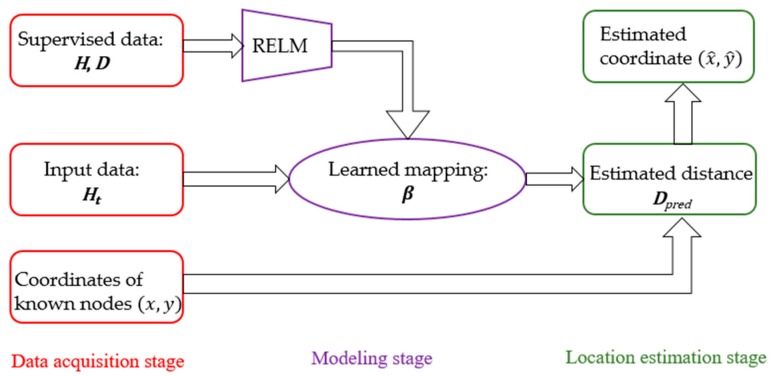
The framework of proposed algorithm. The mapping is firstly trained by REML, using supervised data consist of the known hop-counts and physical distances. After that, in the testing phase, the physical distances of the unknown node are predicted by the learned mapping.

We aim to design a learning machine which transforms the hop-counts to the physical distances. Therefore, we utilize the supervised information (hop-counts and physical distances between anchors) to train a model, including hop-counts and physical distances of the anchor nodes. Then the learned model can be used to predict the physical distances via given hop-counts (from the unknown node to the anchor node). The pipe line of our algorithm is summarized in Figure 4.

The localization of nodes based on ML-RELM method can be divided into three stages: data acquisition, modeling and location estimation. In the data acquisition stage, the program collects the hop-counts and physical distance information between nodes and provides them to the modeling stage. In the modeling stage, the mapping relation between the hop-counts and the physical distances are trained by the hop-counts and physical distances information collected in the data acquisition stage. In the location estimation stage, the distance from the unknown node to the known node is estimated by using the mapping model obtained in the training stage, and the location of the unknown node can be calculated. Details are as follows.

### 4.1. Data Collection

Suppose that *n* nodes are distributed in a two-dimensional plane. The first *m* (*m* << *n*) anchor nodes ${\left\{S_{i}\right\}}_{i=1}^{m}$ are the known self-coordinate information, and the remaining *n−m* nodes ${\left\{S_{i}\right\}}_{i=m+1}^{n}$ are the normal nodes waiting to acquire the location information. When the program begins, any anchor node in the area firstly uses the distance vector routing protocols to broadcast a “HELLO” packet to the remaining nodes. After a period of time, the shortest hop-counts vector ***h****_i_* = [*h_i_*_1_,*h_i_*_2_,…,*h_im_*]^T^ of the anchor node *S_i_* to the remaining anchor nodes can be obtained, and the shortest hop-counts vector ***h****_k_* = [*h_k_*_1_,*h_k_*_2_,…,*h_km_*]^T^ from the unknown node ${\left\{S_{i}\right\}}_{i=m+1}^{n}$ to the anchor node can be obtained. Thus, the shortest hop-counts matrix corresponding to the anchor node and the unknown node is ***H*** = [***h***_1_,***h***_2_,…,***h****_m_*] and ***H****_t_* = [***h****_m_*_+1_,***h****_m_*_+2_,…,***h****_n_*], respectively. The straight distance of the anchor nodes *S_i_* to *S_j_* can be expressed by Equation (6):

(6) $$\begin{array}{l} d\left(S_{i},S_{j}\right)=d_{ij}={\left\|cor\left(S_{i}\right)-cor\left(S_{j}\right)\right\|}_{2}\\ \;\;\;=\sqrt{{\left(x_{i}-x_{j}\right)}^{2}+{\left(y_{i}-y_{j}\right)}^{2}}, \end{array}$$

where $cor\left(S_{i}\right)=\left(x_{i},y_{i}\right)$ is the coordinate of node $S_{i}$, and $d_{ij}$ is the physical distance of the i-th node to the j-th node.

### 4.2. The Proposed Model

According to [9], it is clear that in an ideal case $d\propto w^{T}h$, when each $S_{i}\in B$ collects the *m* pair of data pairs ${\left\{h_{k},d_{i,k}\right\}}_{k=1}^{m}$. Therefore, we assume that the input dimension of ELM is *m*, and the optimal relationship between the physical distance and the optimal relationship between the physical distance and the hop count can be obtained by minimizing the mean square error, that is:

(7) $$\underset{\beta }{\min}\sum_{i=1}^{m}{\left\|e_{i}\right\|}_{2}^{2}=\sum_{i=1}^{m}{\left\|d_{i}-\beta_{i}G\left(h_{i}\right)\right\|}_{2}^{2}$$

where ***d****_i_* = [***d**_i_*_1_,***d**_i_*_2_,…,***d**_im_*]^T^ is a vector which presents the physical distance between the *i-*th anchor node and the remaining ***m***−1 anchor nodes.

Based on the rule of minimum norm solution (ensure both ${\left\|d_{i}-\beta_{i}G\left(h_{i}\right)\right\|}_{2}^{2}$ and ${\left\|\beta_{i}\right\|}_{2}^{2}$ minimum at the same time), Equation (7) exists a minimal norm least squares solution as follows:

(8) $$\beta =G^{†}D,$$

where ***G***^†^ is the augmented inverse of the Moore-Penrose of the hidden layer response matrix and the *i*-th column of ***G*** is the output of the *i*-th hidden node with respect to the input hop vector ***h****_i_*. ***G***^†^ has a variety of calculation methods. In the extreme learning machine, the orthogonal method is often used for the calculation of ***G***^†^: when ***G***^T^***G*** is not singular, ***G***^†^ = (***G***^T^***G***)^−1^***G***^T^; when ***GG***^T^ is not singular, $G^{†}=G^{T}{\left(G^{T}G\right)}^{-1}$.

Since the vector between the hops matrix is prone to correlation, we add a small positive number to the diagonal of ***G***^T^***G*** or ***GG***^T^ when calculating ***G***^†^, making the whole system more stable. Then the hops-distance optimal relation model $\hat{\beta }$ is obtained, e.g., the feedback of single hidden layer is completed. Finally, the program broadcasts the mapping model to each node in the network through distance vector protocol.

The model obtained by RELM actually stores the distance feature of all anchor nodes in all directions, so the model can accurately describe the anisotropic relationship between the hop-count and the physical distance of the nodes. Algorithm 1 summarizes the steps of the model building by RELM algorithm.

**Algorithm 1.** Localization Model Building by RELM.1. Input: the shortest hop-counts matrix $H=\left[h_{1},\ldots ,h_{m}\right]$; the physical distance matrix $D=\left[d_{1},\ldots ,d_{m}\right]$, the count of hidden nodes r. 2. **For**
i from 1 to r 3.  assign input weights $\omega_{i}$ randomly; 4.   assign hidden layer bias $b_{i}$ randomly; 5. **End** 6. **For**
i from 1 to m   calculate the output of hidden layer $G\left(h_{i}\right)$; 7. **End** 8. Calculate the inverse matrix $G^{†}={\left(G^{T}G+kI\right)}^{-1}G^{T}$. 9. Calculate $\hat{\beta }=G^{†}D$. 10. **Output:** the hidden layer weights $\hat{\beta }$.

### 4.3. Location Estimation

For any unknown node, it can obtain the hop-counts vector $h_{k}\subset H_{t}$ and the mapping model $\hat{\beta }$ of the anchor node in the data collection stage to estimate the linear distance from $S_{k}$ to the anchor node, that is:

(9) $$D_{pred}=G\left(H_{t}\right)\hat{\beta }.$$

Then, the unknown nodes combine the coordinates of the anchor nodes and the distance between them to obtain the final coordinates. That is solving the following equations group fork∈{m+1,m+2,…,n}:

(10) $$\left\{ \begin{array}{l} {\left({\hat{x}}_{k}-x_{1}\right)}^{2}+{\left({\hat{y}}_{k}-y_{1}\right)}^{2}={d_{k1}}^{2}\\ \ldots \\ {\left({\hat{x}}_{k}-x_{m}\right)}^{2}+{\left({\hat{y}}_{k}-y_{m}\right)}^{2}={d_{km}}^{2} \end{array}\right.,$$

in which $d_{kj}=D_{pred}\left(k,j\right)$ is the physical distance predicated by Equation (9). Above equations group is usually solved via the maximum likelihood estimation (MLE) of multiple-source location [25]. Specifically, the optimal solution to Equation (10) is expressed as:

(11) $${\hat{c}}_{k}=\left({\hat{x}}_{k},{\hat{y}}_{k}\right)={\left(A^{T}A\right)}^{-1}A^{T}b,$$

where the two projection matrices are:

(12) $$A=2\left[\begin{array}{cc} {\left(x_{1}-x_{m}\right)}^{T}, & {\left(y_{1}-y_{m}\right)}^{T}\\ \ldots & \ldots \\ {\left(x_{m-1}-x_{m}\right)}^{T}, & {\left(y_{m-1}-y_{m}\right)}^{T} \end{array}\right],$$

and:

(13) $$b=\left[\begin{array}{c} {\left(x_{1}-x_{m}\right)}^{2}+{\left(y_{1}-y_{m}\right)}^{2}+d_{km}^{2}-d_{k1}^{2}\\ \vdots \\ {\left(x_{m-1}-x_{m}\right)}^{2}+{\left(y_{m-1}-y_{m}\right)}^{2}+d_{km}^{2}-d_{k1}^{2} \end{array}\right]$$

respectively. Finally the details of location estimation are shown in Algorithm 2.

**Algorithm 2.** Location estimation.1. Input: the shortest hop-counts for unknown nodes to anchor nodes $H_{t}={\left[h_{m+1},h_{m+2},\cdots ,h_{n}\right]}_{m\times \left(n-m\right)}$; the coordinates of known nodes ${\left\{cor\left(S_{i}\right)=\left(x_{i},y_{i}\right)\right\}}_{i=1}^{m}.$. 2. Employ the learned weights of Algorithm 1 to predicate the distances $D=G\left(H_{t}\right)\hat{\beta }$. 3. **For**
k from m+1 to n 4.  calculate matrix A using Equation (12); 5.  calculate matrix b using Equation (13); 6.  calculate ${\hat{c}}_{k}={\left(A^{T}A\right)}^{-1}A^{T}b$; 7. **End**. 8. **Output:** estimated coordinates of unknown nodes ${\left\{\hat{cor}\left(S_{k}\right)={\hat{c}}_{k}=\left({\hat{x}}_{k},{\hat{y}}_{k}\right)\right\}}_{k=m+1}^{n}$

## 5. Performance Evaluation

To analyze and evaluate the performance of the ML-RELM algorithm, we conducted simulation and realistic scenario experiments. The simulation experiments are carried out on a PC (equipped with Windows 7 OS, Intel Core I7 and 8 GB RAM), and all the codes are implemented in MatLab. Realistic scenario experiments are implemented in indoor and outdoor environments respectively.

In order to reduce the one-sidedness of the experimental results in the simulation experiments, each simulation experiment is carried out 50 times. Each node will be randomly re-distributed in the experimental area, taking 50 times average of the Root Mean Square (RMS) [26] as the basis for evaluation:

(14) RMS=1Nt∑i=1Nt((x^i−xi)2+(y^i−yi)2)

where $\left({\hat{x}}_{i},{\hat{y}}_{i}\right)$ is the estimated coordinate of the node. $\left(x_{i},y_{i}\right)$ is the actual coordinate of the i node, and $N_{t}$ is the number of nodes that can be located. In addition, our performance comparisons are implemented among those schemes, including the DV-hop, PDM, LSVR in this section. Since the PDM method needs to set the threshold of TSVD to discard the eigenvalue; the LSVR method needs to set the kernel parameters, the penalty coefficient *C* and the width of insensitive loss function ε. For the sake of fairness, in the experiment, the TSVD method abandons the eigenvectors whose eigenvalues are less than or equal to 2. The *C* and ε of the LSVR method follows the settings in reference [8], and the kernel function is chosen to be the Gaussian kernel function. The kernel function is related to the distance of the training samples, so the kernel parameter is set to 40 times the mean value of the training samples.

### 5.1. Simulation Experiments

Two strategies of the node deployment are considered in the experiment: regular deployment and random deployment. We assume that the nodes are uniformly deployed in the detected region. Our work focuses on anisotropy network. The letter-shaped topology is most commonly used to compare and verify the localization accuracy [4,7,8,18,21]. In order to give a fairly comparison with the classical methods, such as DV-hop, PDM and LSVR, we adopt the same network topologies in the simulation.

#### 5.1.1. Regular Deployment

**Figure 5:**
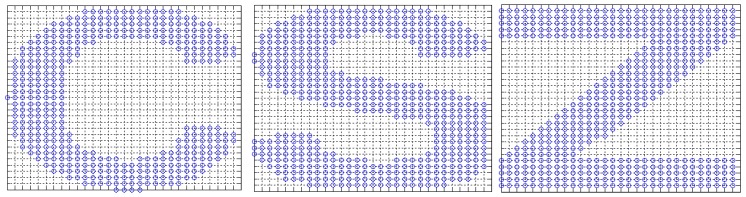
The regular distribution of nodes.

In this group of experiments, as shown in Figure 5, 405, 487 and 525 nodes are regularly deployed in the 600 × 600 C-shaped, Z-shaped and S-shaped areas.

Experiments select 30 to 60 nodes as anchor nodes. First, we examine the final localization results of the two ML-RELM methods (the number of anchor nodes in Figure 5 is 40), where the circle represents the unknown node, and the square represents the anchor node. Then, as shown in the Figure 5, we use a straight line to connect the real coordinates of the unknown node and its estimated coordinates. The longer the line, the greater the estimation error of nodes.

**Figure 6:**
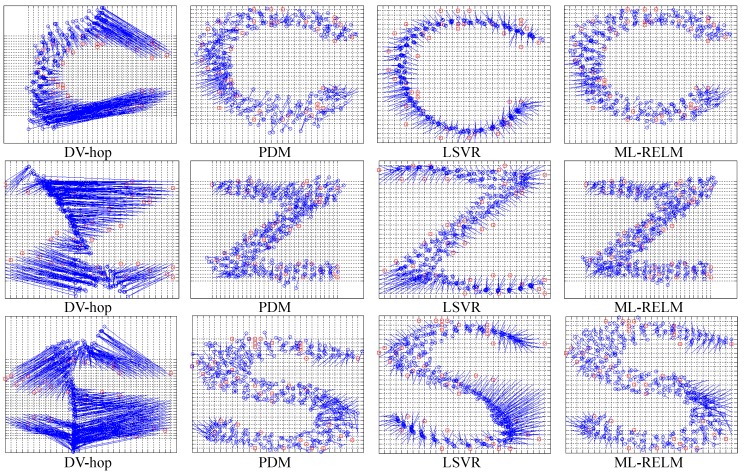
The localization results of regularly distributed nodes.

It is obvious to observe from Figure 6 that the DV-hop method uses fixed hop-counts vs distances coefficients, and when there is an obstacle, the hop-count and the distance may deviate on converting. Although the PDM method can solve hop-distance deviation to a certain extent, it cannot deal with significantly different scales. For LSVR, the optimization of multiple parameters easily results in over-fitting (unknown nodes estimate an unknown trend to a curve). Nevertheless, estimation accuracy from the proposed ML-RELM is higher than from other localization methods.

**Figure 7:**
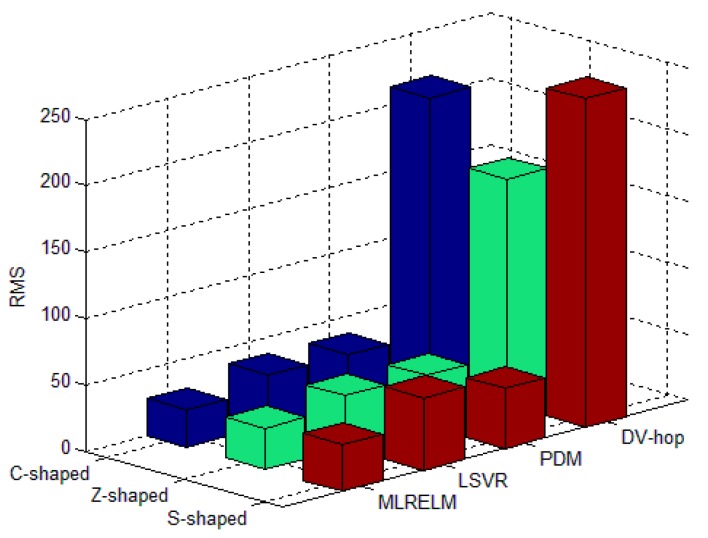
Average RMS for different algorithms in the regular deployment environment.

For each deployment area in the regular deployment network, Figure 7 shows the average RMS value for 50 experiments with 40 anchor nodes. The experimental results illustrated in Figure 7 show that the classical multi-hop localization method in the C-shaped, Z-shaped and S-shaped regions has low DV-hop performance, and the RMS values are 214.22, 169.75 and 246.65, respectively. The PDM and LSVR methods have better ability to obtain the relationship between the hop-counts and distances, so their performance is improved by 80.9% and 79.2% compared with the DV-hop method. ML-RELM has improved the prediction and fitting ability by using ELM as the core algorithm, so the ML-RELM localization performance is improved by 21.8% and 28.3% respectively compared with the PDM and LSVR methods.

The ELM machine is very efficient, which only needs to calculate the weights (the computation complexity is $O\left(\min\left(L^{3},N^{3}\right)\right)$). So the ELM is often in a very short period of time able to complete the operation. Table 1 shows that the running time of our proposed ML-RELM algorithm is compared with that of multiple multi-hop distributed localization algorithms in a variety of regular deployment environments of 40 anchor nodes. Clearly, ML-RELM is proposed to be more efficient than LSVR, DV-hop and PDM.

**Figure 8:**
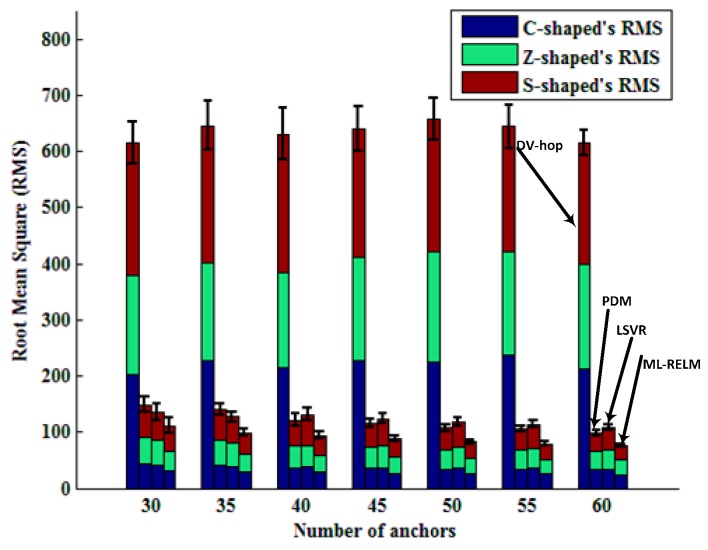
Comparison of different algorithms with different number of anchor nodes in the regular deployment.

Bars in Figure 8 represent RMS values and standard deviations of the four algorithms varying with the number of anchor nodes in the three regular deployment areas of the C, Z, and S shapes. It is easy to see that DV-hop is not suited to non-convex environment (there are obstacles in the network) localization. We also notice that the LSVR algorithm is superior to PDM when the number of anchor nodes is less than 40, and the PDM algorithm is better than LSVR. This is due to the increase in the number of anchor nodes, resulting in the over-fitting, making the performance of LSVR degraded (shown in Figure 6).

#### 5.1.2. Random Deployment

**Figure 9:**
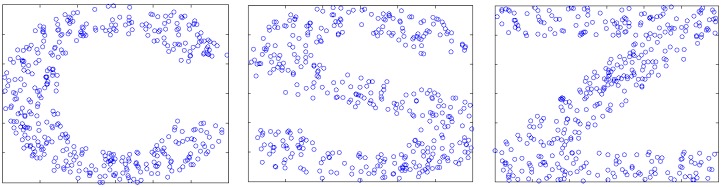
The random distribution of nodes.

In this group of experiments, 400 nodes are randomly deployed in 600 × 600 two-dimensional square areas, due to the reasons of the block, the deployment shaped to C, S and Z-type (shown in Figure 9).

**Figure 10:**
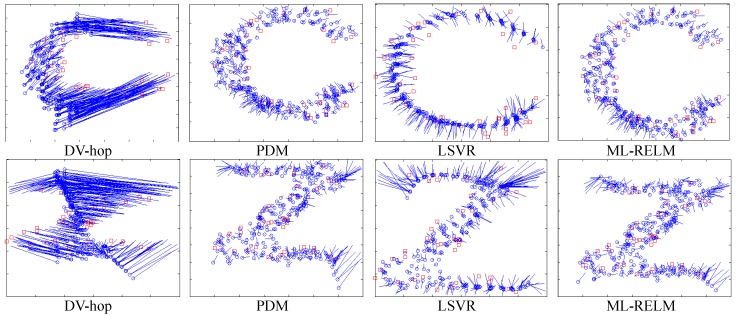

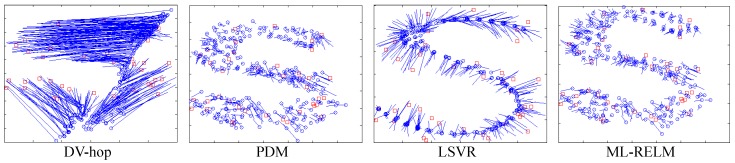
The localization results of randomly distributed nodes.

As shown in Figure 10, we examine the final localization result of a set of ML-RELM methods (the number of anchor nodes is 40). It is clear from the figure that the proposed method performance is still superior to other localization methods.

**Figure 11:**
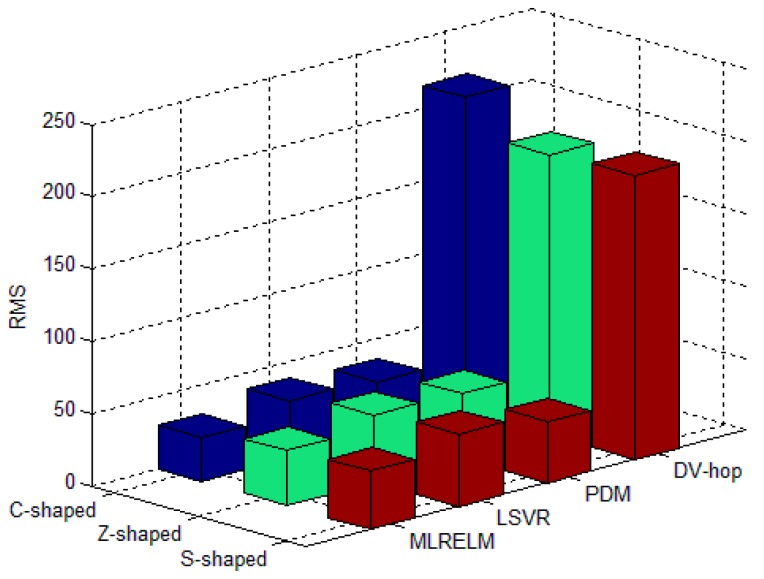
Average RMS for different algorithms in the random deployment environment.

After 50 trials, the average RMS values of the four localization algorithms in the three type areas are shown in Figure 11. The proposed ML-RELM performance is 82.2%, 12.1%, and 20.5% higher than that of DV-hop, PDM, and LSVR respectively.

Table 2 describes the running time of four algorithms in three random deployment environment. The ML-RELM method is more efficient and superior to the latest LSVR method.

**Figure 12:**
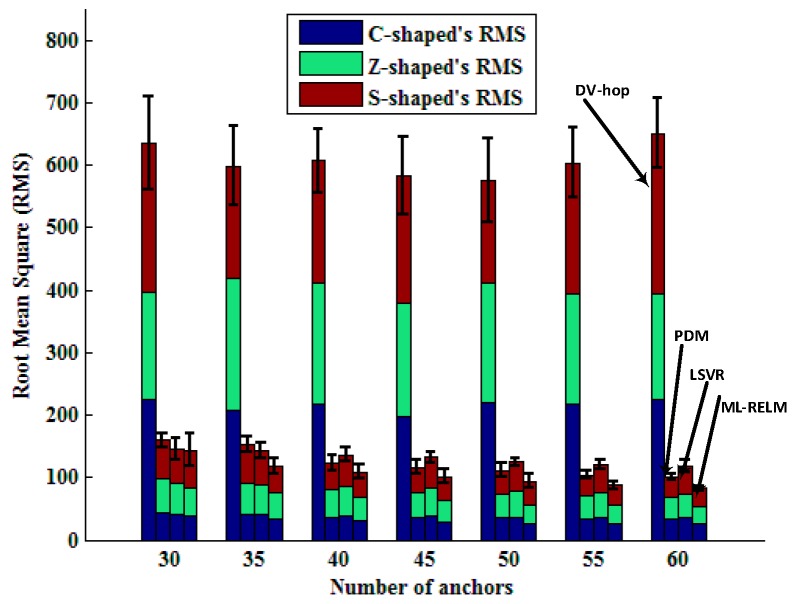
Comparison of different algorithms with different number of anchor nodes in the random deployment.

Bars in Figure 12 represent RMS values and standard deviations of the four algorithms varying with the number of anchor nodes in the three random deployment areas of the C, Z, and S shapes. It is also obvious that ML-RELM has the best performance, and LSVR is degraded due to over-fitting when the anchor nodes numbers are large.

### 5.2. Realistic Scenario Experiments

**Figure 13:**
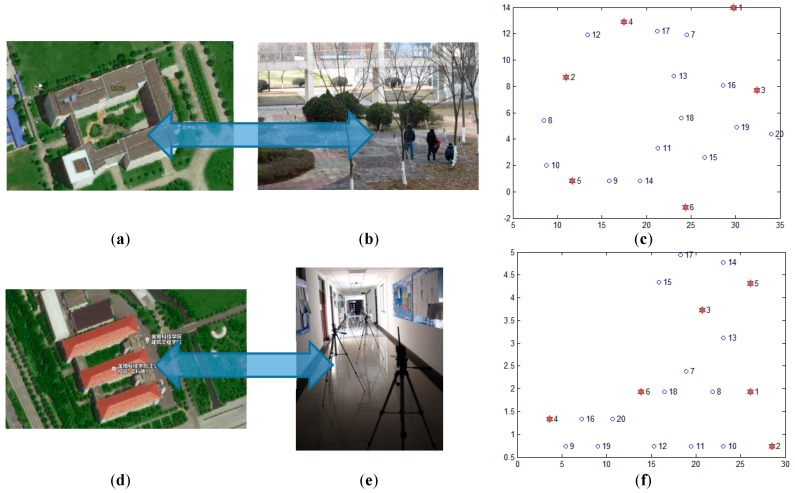
Nodes distribution under realistic scenarios (**a**) Satellite picture of outdoor location; (**b**) Picture of outdoor test-bed; (**c**) Topological graph of outdoor test-bed; (**d**) Satellite picture of indoor location; (**e**) Picture of indoor test-bed; (**f**) Topological graph of indoor test-bed.

In order to evaluate the performance of the proposed algorithm ML-RELM in realistic situations, we employ 20 nodes with the TI CC2530 chip which are deployed outside of buildings (as shown in Figure 13b) and inside of buildings (as shown in Figure 13e) respectively at the Jinling Institute of Technology. Figure 13a,d are satellite images of the test locations, respectively. The size of the outdoor and indoor test-beds is 30 m × 5 m and 30 m × 16 m, respectively. We set the communication radius of nodes equal to 6 m. Nodes 1#, 2#, 3#, 4#, 5# and 6# are selected as anchor nodes, and the remaining 14 nodes are normal nodes. Other parameters are similar to that in simulation experiments. Three previous methods: (1) The classic DV-hop algorithm [10]; (2) PDM [9]; and (3) LSVR [7,8] are compared. 

**Figure 14:**
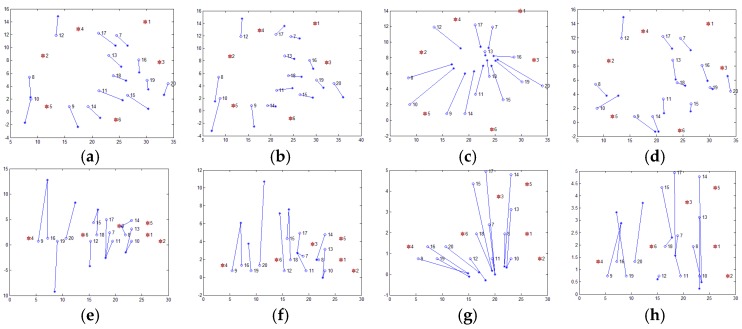
Results of location estimation in realistic scenarios (**a**) DV-hop in the outdoor, RMS = 3.1446; (**b**) PDM in the outdoor, RMS = 3.0085; (**c**) LSVR in the outdoor, RMS = 5.7151; (**d**) ML-RELM in the outdoor, RMS = 2.6829; (**e**) DV-hop in the indoor, RMS = 6.6422; (**f**) PDM in the indoor, RMS = 4.4114; (**g**) LSVR in the indoor, RMS = 5.0065; (**h**) ML-RELM in the indoor, RMS = 2.5197.

As shown in Figure 14, the ML-RELM method proposed in this paper can achieve higher localization accuracy under these two conditions. The average RMS of ML-RELM is about 2.4 outdoor. Compared with DV-hop, PDM and LSVR, the average performance of ML-RELM is improved by 17.93%, 12.78% and 51.55%, respectively. Besides, the average RMS of ML-RELM is about 2.3 indoor. Compared with DV-hop, PDM and LSVR, the average localization accuracy (measured by the RMS) is 61.27%, 39.99% and 45.58% higher than DV-hop, PDM and LSVR respectively.

**Figure 15:**
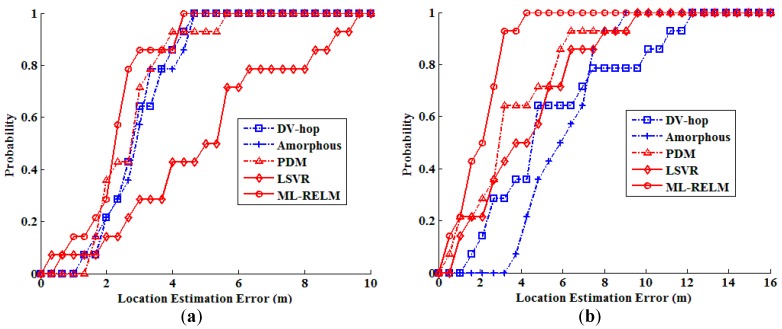
The Cumulative Distribution Function (CDF) of the errors in realistic scenarios (**a**) CDF of outdoor; (**b**) CDF of indoor.

In these two group of realistic scenario experiments, we also study the Cumulative Distribution Function (CDF) of the localization errors. CDF shows the percentage of localized nodes whose localization error is smaller than some thresholds, thus tells us whether local damages have proliferated to other areas. Figure 15 shows the CDF of localization errors outdoor and indoor. We can find that when using ML-REML the localization performance is effectively improved. More than 71% nodes are localized with localization error less than 3 m outdoor, and more than 64% nodes are localized with localization error less than 3 m indoor.

## 6. Conclusions

A novel large-scale range-free localization algorithm for wireless networks is proposed in this paper. The proposed method is based on regularized extreme learning, which is a type of quick speed and less intervention single-hidden-layer feedforward neural network, one of the newest machine learning algorithms. In the proposed algorithm, each node estimates its location using the model by regularized extreme learning trained with anchor nodes. In this model, the location of the node is predicted by the information related to the shortest hop-counts of the node to all of the anchor nodes. The proposed algorithm is applied to the complex deployment environment. The experimental results show that the proposed algorithm is more efficient and accurate than the existing multi-hop localization methods in a variety of deployment environments. 

## Figures and Tables

**Table 1:** Running time in the regular deployment.

CPU TIME (m)		DV-Hop	PDM	LSVR	ML-RELM
40 anchors, 365 unknown nodes (C-shaped)	**Average**	1.0917	1.1244	1.5707	1.1027
	**Median**	1.0782	1.1081	1.5586	1.0858
	**Worst**	1.2618	1.3289	1.679	1.2232
	**Standard deviation**	0.0517	0.0697	0.0445	0.0456
40 anchors, 447 unknown nodes (Z-shaped)	**Average**	2.2876	2.3103	2.7801	2.2796
	**Median**	2.3745	2.3779	2.8577	2.3926
	**Worst**	2.9239	2.7247	3.1042	2.5051
	**Standard deviation**	0.1730	0.1746	0.1933	0.1870
40 anchors, 485 unknown nodes (S-shaped)	**Average**	2.4172	2.4079	2.8511	2.3901
	**Median**	2.3745	2.3779	2.8577	2.3926
	**Worst**	2.9239	2.7247	3.1042	2.5051
	**Standard deviation**	0.1534	0.1063	0.1024	0.0696

**Table 2:** Running time in the random deployment.

CPU TIME (m)		DV-Hop	PDM	LSVR	ML-RELM
40 anchors, 360 unknown nodes (C-shaped)	**Average**	1.0561	1.09	1.5637	1.0852
	**Median**	1.0588	1.0854	1.5563	1.0855
	**Worst**	1.1127	1.1391	1.7832	1.1334
	**Standard deviation**	0.0245	0.0235	0.0719	0.0320
40 anchors, 360 unknown nodes (Z-shaped)	**Average**	1.0901	1.1048	1.5693	1.0913
	**Median**	1.0805	1.0981	1.5701	1.0839
	**Worst**	1.2112	1.1631	1.6103	1.1226
	**Standard deviation**	0.0376	0.0242	0.0189	0.0181
40 anchors, 360 unknown nodes (S-shaped)	**Average**	1.0201	1.0346	1.4501	1.0256
	**Median**	1.0185	1.0343	1.4793	1.0287
	**Worst**	1.0665	1.0657	1.5043	1.0518
	**Standard deviation**	0.0285	0.0185	0.1173	0.0219
